# A narrative review on the similarities and dissimilarities between myalgic encephalomyelitis/chronic fatigue syndrome (ME/CFS) and sickness behavior

**DOI:** 10.1186/1741-7015-11-64

**Published:** 2013-03-08

**Authors:** Gerwyn Morris, George Anderson, Piotr Galecki, Michael Berk, Michael Maes

**Affiliations:** 1 Tir Na Nog, Bryn Road Seaside 87, Llanelli, SA152LW, UK; 2 CRC Clinical Research Centre/Communications, Laurel Street 57, Glasgow, G11 7QT,UK; 3 Department of Adult Psychiatry, Medical University of Lodz, Aleksandrowska 159, Lodz, 91229, Poland; 4 Barwon Health, School of Medicine, Deakin University, PO Box 291, Geelong, 3220, Australia; 5 Orygen Youth Health Research Centre, Poplar Road 35, Parkville, 3052, Australia; 6 Centre of Youth Mental Health, University of Melbourne, Poplar Road 35, Parkville, 3052, Australia; 7 The Florey Institute for Neuroscience and Mental Health, University of Melbourne, Kenneth Myer Building, Royal Parade 30, Parkville, 3052, Australia; 8 Department of Psychiatry, University of Melbourne, Level 1 North, Main Block, Royal Melbourne Hospital, Parkville, 3052, Australia; 9 Department of Psychiatry, Chulalongkorn University, Rama 4 Road 1873, Pathumwan, Bangkok, 10330, Thailand

**Keywords:** CFS, chronic fatigue, depression, inflammation, ME, oxidative stress, sickness behavior

## Abstract

It is of importance whether myalgic encephalomyelitis/chronic fatigue syndrome (ME/CFS) is a variant of sickness behavior. The latter is induced by acute infections/injury being principally mediated through proinflammatory cytokines. Sickness is a beneficial behavioral response that serves to enhance recovery, conserves energy and plays a role in the resolution of inflammation. There are behavioral/symptomatic similarities (for example, fatigue, malaise, hyperalgesia) and dissimilarities (gastrointestinal symptoms, anorexia and weight loss) between sickness and ME/CFS. While sickness is an adaptive response induced by proinflammatory cytokines, ME/CFS is a chronic, disabling disorder, where the pathophysiology is related to activation of immunoinflammatory and oxidative pathways and autoimmune responses. While sickness behavior is a state of energy conservation, which plays a role in combating pathogens, ME/CFS is a chronic disease underpinned by a state of energy depletion. While sickness is an acute response to infection/injury, the trigger factors in ME/CFS are less well defined and encompass acute and chronic infections, as well as inflammatory or autoimmune diseases. It is concluded that sickness behavior and ME/CFS are two different conditions.

## Introduction

Humans and animals use a range of autonomic, metabolic and behavioral responses to combat acute infections or injuries. Sickness behavior is a physiological behavioral response principally induced and regulated by proinflammatory cytokines, including interleukin 1β (IL-1β), IL-6 and tumor necrosis factor (TNF)α, which act centrally to induce sickness behaviors, including pyrexia. Proinflammatory cytokines modify the activity of hypothalamic neurons causing an increase of the thermoregulatory set point [1]. In addition, animals and humans display symptoms such as fatigue, malaise, hyperalgesia, neurocognitive disorders, sleepiness, anhedonia, anorexia and weight loss, as well as diminished food intake, social activities, locomotor activity, grooming and exploration [2,3]. Sickness behaviors are viewed as adaptive responses, which facilitate recovery from acute infections or injuries [2,3]. Sickness behaviors, including fever, are postulated to offer a survival benefit in endothermic and ectothermic vertebrates [4]. The sickness response is conserved by evolution and has an adaptive function in enabling survival of individuals and species in the face of assault by a myriad of microbial pathogens and injuries [5]. Stereotypical patterns of sickness are displayed by mammals, birds, reptiles and even invertebrates [6]. In summary, (a) this proinflammatory cytokine-induced sickness response is short lasting, evolutionarily conserved, and beneficial to the organism; and (b) many of the sickness behaviors are valuable in conserving energy and therefore to combat and attenuate the inflammatory response caused by infections or inflammatory trauma [3,7].

Myalgic encephalomyelitis/chronic fatigue syndrome (ME/CFS) is a disabling disorder that can have a greater impact on functional status and well-being than many other chronic, life-threatening diseases [8,9]. ME/CFS is associated with a dramatic decrement in physical functioning [10] with patients scoring significantly lower on most of the eight Medical Outcome Study (MOS) 36-item Short-Form (SF-36) health survey subscales when compared with patients with cardiovascular and neurological disorders, as well as higher on depression measures [8]. Typical symptoms of ME/CFS are: physical and mental fatigue, pain, muscle weakness, neurocognitive impairment, sleep disturbances, depression, gastrointestinal symptoms, a subjective feeling of infection or a flu-like malaise and post-exertion fatigue or malaise [11,12].

Thus, there are important behavioral/symptomatic similarities between both ME/CFS and sickness behavior. Moreover, activated immunoinflammatory pathways play a role in the pathophysiology of ME/CFS, for example, increased proinflammatory cytokine levels [13]. As such, both sickness behavior and ME/CFS share common phenomenological and biochemical aspects. These findings led some authors to conclude that ME/CFS and sickness behaviors are a manifestation of a common pathophysiology with other neurophysiological mechanisms of sickness behavior being similarly extrapolated to ME/CFS and related conditions [14]. Other authors maintain that ME/CFS is fundamentally a more chronic version of acute sickness behavior [15]. The latter authors in fact change the meaning of the term sickness behavior and use the term to describe a situation where the sickness behavioral complex and the inflammatory condition persist. This situation should then be recognized as persistent sickness behavior or ME/CFS.

A further confounding factor is the diagnosis and subtyping of ME/CFS. There are different diagnostic criteria used to make the diagnosis of ME/CFS, generating considerable controversy. According to a commonly used diagnostic classification for ME/CFS, Fukuda's criteria [16], ME/CFS is accompanied by chronic fatigue lasting for more than 6 months and at least four additional symptoms (sore throat, tender lymph nodes, neurocognitive disorders, multiple joint pain, muscle pain, headache, non-refreshing sleep or post-exertion malaise (PEM) lasting more than 24 h). The diagnosis of ME/CFS according to Fukuda's criteria, however, defines a heterogeneous group of patients [11]. Recently, we have provided evidence suggesting that ME/CFS patients should be divided into those with and without post-exertion malaise into ME and CFS, respectively. Doing so, it was defined that ME and CFS are qualitatively distinct diagnostic classes [11].

Another major confound is the common perspective within psychosocial psychiatry to consider ME/CFS as a condition triggered by excessive rest in predisposed individuals following acute triggers [17]. This perspective places ME/CFS within a psychosocial and psychiatric framework. Recently, we have incorporated aspects of this within a biological model with psychosocial aspects contributing to, and themselves being driven by, inflammatory and related pathways, providing a more parsimonious explanation based on biological underpinnings [17].

In the present work, we review: (a) the phenomenological similarities and dissimilarities between sickness behavior and ME/CFS, (b) the course of sickness behavior versus ME/CFS, (c) the immunoinflammatory-related pathways that underpin or may discriminate both sickness and ME/CFS, and (d) the differences in etiologic factors that trigger both conditions.

## Phenomenological similarities between sickness behavior and ME/CFS

Symptoms typical of sickness behavior are described above. The phenomenological experience of acute viral or bacterial infection involves malaise, a lack of motivation or lassitude, with accompanying fatigue bordering on exhaustion. Other responses include numbness, shivering, impaired appetite and weight loss, as well as aches and pains in muscles and joints [3].

Table 1 summarizes the similarities and dissimilarities between sickness behavior and ME/CFS. People with ME/CFS have a wide range of classically conceptualized psychosomatic, but recently reframed physiosomatic, symptoms [11-13]. Typical symptoms include fatigue and chronic exhaustion. These symptoms include unrelenting severe disabling mental and physical fatigue combined with disabling levels of muscle fatiguability. Hyperalgesia, muscle pain and tension, migraine type headaches, insomnia, hypersomnia, non-refreshing sleep or sleep wake cycle reversal are typical symptoms of ME/CFS. Problems with memory retrieval can manifest as an inability to finish sentences, negatively feeding back on self-esteem and subjective wellbeing. These symptoms are exacerbated by cognitive and or physical activity. Intolerance to even trivial increases in physical or mental activity above individual norms is the hallmark symptom of ME [10-12,17,18]. This intolerance manifests itself as marked worsening of symptoms that may be short lived or prolonged (sometimes weeks) but only rarely resulting in permanent disability [10,11]. Post-exertion malaise indicates the intense malaise, exhaustion or neurocognitive symptoms following physical or mental activities and lasting for more than 24 h. This typical symptom in ME corresponds with the fatigue and exhaustion behaviors expressed during sickness behavior.

**Table 1:** Characteristics of myalgic encephalomyelitis/chronic fatigue syndrome (ME/CFS) and sickness behavior

Category	ME/CFS	Sickness behavior
Physiosomatic symptoms	Disabling fatigue	Fatigue, lethargy, behavioral inhibition
	Mental fatigue	Reduction of exploration
	'Pacing' as an energy-conservation strategy	Reduced locomotor activity
	Post-exertion malaise following mental/physical activities	Fatigability
	A flu-like malaise	Malaise, flu-like symptoms
	Hyperalgesia	Hyperalgesia
	Muscle tension and pain	Muscle pain
	Sleep disorders	Sleepiness
	High incidence of autonomic symptoms	Probably yes, but not well documented
	Failure to concentrate	Failure to concentrate
	Memory disturbances	Memory disturbances
	Gastrointestinal symptoms	-
Depressive symptoms	May occur when comorbid depression is present	Disinterest in social interactions
	Anhedonia may occur when depression co-occurs	Anhedonia, or reduced intake of sweetened milk in rodent models
	Sadness	Sadness
Anorexia/weight loss	May occur when comorbid depression is present	Anorexia and weight loss
Pyrexia	Slightly increased body temperature in a few patients	Pyrexia
Onset	Acute onset or insidious	Acute onset
Course	Waxing and waning or progressive course	Acute adaptive response
	Chronic course (>6 months)	Maximal 19 to 43 days
Energy metabolism	Mitochondrial dysfunction, lowered ATP, abnormally high lactate levels	Is an adaptive behavioral response aiming to conserve energy and to redirect energy to immune cells to combat the pathogens
		Is an adaptive response to counteract negative energy balance
	Impaired oxidative phosphorylation	Sickness behavior plays a key role in the resolution of acute inflammation
		When the energy stores are depleted and the acute inflammation is not resolved, chronic inflammation ensues
	Structural mitochondrial abnormalities	
	Accelerated glycolysis; decreased phosphocreatine synthesis rates following exercise	
Pathways	(Sub)chronic inflammation with increased proinflammatory cytokines	Acute inflammation with increased proinflammatory cytokines
	Cell-mediated immune (CMI) activation	Probably activated
	Simultaneous T helper (Th)1 and Th2 responses	-
	Multiple immune dysfunctions	-
	Lowered antioxidant levels	-
	Reactive oxygen species (ROS)/reactive nitrogen species (RNS)	Probably yes
	Damage by oxidative and nitrosative stress (O&NS) to lipids, DNA, proteins	-
	Autoimmune responses to O&NS modified neoepitopes	-
	Autoimmunity	-
	Reduced hypothalamic-pituitary-adrenal (HPA) axis function in some patients	Enhanced HPA axis activity (part of compensatory (anti)-inflammatory reflex system (CIRS))
Triggers	Multiple, not well defined	Acute, highly defined
	Long-term effects of acute infection	Acute pathogens and tissue injury
	Disease exacerbated by infections	-
	Disease exacerbated by psychological stress	-
	Chronic medical inflammatory illness	-
	Chronic neuroinflammatory disorders	-
	Autoimmune disorders	-
	Sometimes no trigger factor is observed	Is always a response to a defined trigger
Risk factors	IgG, IgG1 and IgG3 deficiencies	-
	Immune gene polymorphisms	-
	Reduced ω3/ω6 ratio	-
General	Inflammation, O&NS and mitochondrial-related chronic progressive disorder	Inflammation-induced adaptive behavioral and CIRS response that is conserved through evolution
Janus face	Bad 'chronic' side: a chronic disorder with positive feedback loops between inflammatory responses and autoimmune processes	Beneficial 'acute' side: supports inflammation, redirects energy to immune cells, conserves energy and prevents negative energy balance, helps eradicating the trigger, and has anti-inflammatory effects

Malaise, a key aspect of sickness behavior, is also a major feature of ME/CFS. Many individuals with ME/CFS display symptoms normally associated with severe influenza, for example, including a flu-like malaise or the subjective feeling of infection [11]. This symptom scores highly on two symptomatic dimensions of ME/CFS pathology; that is, a sickness dimension that loads highly on neurocognitive disorders, depression, and autonomic disorders and a hyperalgesia dimension that loads highly on muscle pain and tension and headache. This could be seen as analogous to the malaise theory of depression, which considers malaise as a key feature of depression [7], which we have recently shown could not be validated, as only some depressed patients experience malaise [19]. However, this highlights the phenomenological and biochemical overlaps among depression, somatization and ME/CFS [20].

Depressive-like behaviors, including reduction of locomotor activity, anhedonia (reduced intake of sweetened milk in rodent models), anorexia and weight loss are characteristics of sickness behavior and are not specific to ME/CFS. However, there are high rates of comorbidity between ME/CFS and depression. Subclinical and full-blown clinical depression frequently accompany ME/CFS [21]. Moreover, measures of clinical depression and ME/CFS show high comorbidity, with clinical depression being the most prevalent comorbidity with ME/CFS in some studies [22]. This has led some to propose that ME/CFS is a form of clinical depression [23]. In addition, depressive symptoms, such as sadness, are included in rating scales, such as the Fibromyalgia and Chronic Fatigue Syndrome Rating Scale [24]. Thus, in fact, patients with ME/CFS may have anorexia, weight loss, and so on, when they also have clinical depression. However, clusters of physiosomatic symptoms coupled to increases in immunoinflammatory pathways are significant commonalities in depression and ME/CFS, suggesting significant phenomenological and biochemical similarities that may be relevant to overlaps in subtyping and treatment [20]. Nevertheless, clinical depression and ME/CFS are different syndromes, which may be discriminated with a high predictive value using severity of post-exertion malaise, percentage of time fatigue reported, shortness of breath, unrefreshing sleep, confusion/disorientation and self-reproach [25].

Other differences between sickness behavior and ME/CFS are pyrexia and gastrointestinal symptoms. Pyrexia is one of the key symptoms of sickness behavior and plays a role in the defense against acute infections and acute injury. Mild and moderate fever prevents viral replication and enhances crucial functions of polymorphonuclear leukocytes, including mobility and killing of bacteria [3]. The effects of proinflammatory and cell-mediated immune (CMI) cytokines, including interferons, are more active during fever [3]. There is, however, no evidence of pyrexia in ME/CFS. Nevertheless, the onset of ME/CFS as defined by fatigue, arthralgias, myalgias and a chilly sensation may be accompanied by low-grade pyrexia [26]. In a small proportion of fatigued individuals, fatigue and isolated fever may apparently persist without organic pathology [27]. In contrast to sickness behavior, many patients with ME/CFS have gastrointestinal symptoms as measured by the Fibromyalgia and Chronic Fatigue Syndrome Rating Scale [24]. Gastrointestinal symptoms, reminiscent of irritable bowel syndrome (IBS), are considered to be diagnostic criteria in new ME and ME/CFS case definitions [10,11]. In addition, these gastrointestinal symptoms may have a specific inflammatory pathophysiology, including via increased bacterial translocation [12].

ME/CFS patients display signs of autonomic nervous system dysfunction [28,29] and although not typically described as core symptoms of sickness behavior these symptoms may also occur during acute inflammatory conditions. Neurally mediated hypotension and orthostatic intolerance are the most commonly documented cardiovascular symptoms in ME/CFS. The latter abnormality in particular correlates with the severity of disease [30,31]. Postural orthostatic tachycardia syndrome is another common finding. Exaggerated postural tachycardia and enhanced sympathetic activity have been reported [28]. Intolerance of wide temperature changes and markedly impaired thermostatic stability are other commonly reported manifestations of autonomic dysfunction. A subdued cardiac response to exercise has been reported [32] and sympathetic hyperactivity coupled with reduced vagal modulation are reproducible findings [33]. De Becker *et al*. [34] detected a sympathetic drive mediated increased heart rate on tilt compared to healthy controls. Another study demonstrated impaired heart rate responses to exercise coupled with globally impaired hemodynamic responses incompatible with patient deconditioning or prolonged inactivity [35]. Several other authors have reported autonomic dysfunctions in people with ME/CFS [36-38].

In summary, cross-sectionally there is a phenomenological overlap between sickness behavior and ME/CFS, both presenting with malaise, hyperalgesia, fatigue, exhaustion, sleepiness, failure to concentrate, and sometimes mood disturbances. PEM following mental and physical activities, a characteristic symptom of ME, may also occur during sickness behavior. Other symptoms or behaviors, however, discriminate ME/CFS and sickness behavior. For example, gastrointestinal symptoms reminiscent of IBS [10,11] occur in many ME/CFS patients but not typically in sickness behavior. Anorexia and weight loss, typical symptoms of sickness, are not germane to ME/CFS unless there is comorbid depression. While pyrexia is a hallmark of the acute inflammation during sickness, mild fever may occur in a small proportion of ME/CFS patients. Phenomenologically, the acute sickness response demonstrates some overlap with ME/CFS but the range of symptoms experienced by people with ME/CFS is much wider than in sickness behavior.

## Course: sickness behavior versus ME/CFS

Sickness behavior is conceptualized as a short-term response to acute infections or injuries. Sickness is an adaptive behavioral response that is appropriate to counteract acute bacterial or viral infections or inflammatory trauma and therefore plays a role in the resolution of inflammation and thus recovery. ME/CFS, on the contrary, is an enduring disorder with a relapsing-remitting or chronic course [12,39]. Using international consensus criteria for ME/CFS, most studies report a waxing and waning or progressive pattern of this disease [40-42]. In contrast to sickness behavior, which is a short-lasting, beneficial behavioral response, the recovery rate from ME/CFS is very low [40-42]. For example, in studies using CDC criteria the recovery rate was only 4% [40,42]. In another prospective study carried out over 12 months, none of the patients recovered, while 40% of the individuals with ME/CFS did not improve, 20% showed a progressive course, and 40% showed a relative improvement in their symptoms [43].

Recent use of the terms prolonged, persistent or inappropriately sickness behavior has emerged. Within this terminological perspective, ME/CFS and sickness behavior may be regarded as manifestations of a shared pathophysiology, with ME/CFS viewed as an expression of persistent increases in proinflammatory cytokines following an episode of acute inflammation. We have argued that attaching the terms prolonged, exaggerated or persistent to sickness behavior is unsatisfactory, primarily due to sickness behavior by definition, being a short-lasting, beneficial behavioral response that plays a role in the resolution of inflammation [3]. As discussed above, some authors consider that the sickness responses may persist when the production of proinflammatory cytokines, for example following gut and hepatic inflammation or choleostasis, is no longer restricted to the periphery, but also becomes established systematically, and perhaps prolonged, within the CNS [44-46]. Prolonged fatigue and depression, however, are not generally viewed as adaptive behaviors but are more parsimoniously seen as dysfunctional [7] as is the case in autoimmune disorders or persistent infections. Therefore, these conditions cannot be termed sickness behavior (defined as an acute adaptive response), with these patients more likely to have secondary depression, CFS or ME perhaps as a result of pre-existing genetic or epigenetic priming that alters the longer-term consequences of infection and other stressors.

'Persistence' of inflammation is in our opinion not an adequate term and should better be labeled as 'transition from an acute inflammatory response to a chronic inflammatory state'. The 'transition' label stresses that the pathways underpinning the pathophysiology of chronic inflammation and chronic inflammatory disease are distinct from the beneficial mechanisms that determine sickness behavior [3]. In this respect it was shown that such transition towards chronic inflammatory conditions may play a role not only in ME/CFS but across a range of different diseases, for example, post-trauma illness, malaria, sepsis, and so on [15]. There is a further overlap between sickness behavior as a physiological construct, and abnormal illness behavior as a psychological/behavioral construct. The latter overlaps with illness investment and the sick role, and may be concurrent or overlapping phenomena [47].

In summary, while sickness behavior is a short-lasting, adaptive and acute inflammatory state, ME/CFS is a chronic disorder characterized by a waxing and waning or progressive pattern with an extremely low recovery rate (see Table 1).

## Neuroimmune pathways in sickness behavior and ME/CFS

### Energy metabolism in sickness behavior versus ME/CFS

Acute inflammatory conditions consume large amounts of energy, whereby adipose and muscle tissues and glycogen are used to provide energy to combat pathogens [48]. This causes a negative energy balance; that is, the expended usage of calories is greater than the intake. Lowered synthesis of muscle proteins, proteolysis, and lipolysis and loss of tissue proteins and fats are typically induced by proinflammatory cytokines produced in response to invading pathogens in injured tissues [48]. The objective of the sickness response from an evolutionary perspective is energy conservation [3,7,15]. Proinflammatory cytokines, such as TNFα, not only modulate a negative energy balance to compensate for the increased energy demands but also reduce food intake by causing anorexia and decreased voluntary energy utilization [48]. Characteristic sickness behavior symptoms, such as lethargy, sleepiness, fatigue, listlessness, malaise, hyperalgesia, psychomotor retardation, loss of libido and cognitive deficits aim to limit highly energy-consuming processes, such as motor, sexual and brain activities [3]. As such, energy that otherwise would be consumed for locomotor, reproductive and neurocognitive activities is withdrawn from the brain, muscles and other peripheral tissues and redirected to fuel the calorie-dependent activities of immune cells [2,3,7,15]. Thus, the typical sickness behaviors save energy and contribute to redirecting the conserved energy to immune cells as well as to pyrogenic and other inflammatory processes. As such, sickness behaviors, in shifting resources to a patterned immune response, help to combat acute infection or injury.

Moreover, by inducing anorexia (and thus restricting calorie intake), intracellular signaling pathways associated with inflammation are attenuated. This includes those leading to IL-6 production [49]. Calorie restriction additionally attenuates lipopolysaccharide (LPS)-induced sickness behavior in a dose-dependent manner [50]. Therefore, we have argued that sickness behavior is part of the compensatory (anti)-inflammatory reflex system (CIRS), which is induced in response to inflammatory processes [3]. Thus, sickness behavior augments the beneficial effects of inflammation and at the same time limits an overzealous inflammatory response [3,50]. The increased energy expenditure and anorexia leading to the negative energy balance eventually causes loss of body fat, protein mass and thus lean body mass and therefore may lead to inflammation-associated weight loss [3]. Weight loss results in anti-inflammatory effects attenuating the production of, for example, IL-6 and TNFα and increasing that of endogenous anti-inflammatory compounds, for example, adiponectin [3]. Thus, not only proinflammatory cytokine-induced anorexia but also the consequent weight loss is a homeostatic adaptive response, which should be considered as part of the CIRS [3].

Finally, energy saving sickness behaviors, such as fatigue, listlessness, loss of libido, and neurocognitive disorders, not only play a role in the resolution of inflammation but are crucial in preventing the transition from acute to chronic inflammation. Thus, by saving energy and compensating for the negative energy balance sickness behaviors prevent inflammatory sequelae, such as depletion of energy stores, cachexia, anemia, osteopenia and insulin resistance, which determine the transition towards chronic inflammation [51]. The transition from acute to chronic inflammation occurs around 19 to 43 days after the onset of the acute phase of inflammation [51]. Chronic inflammation ensues when the acute inflammatory response and the CIRS, including sickness behavior, were not able to eradicate the primary infectious agent or heal the injury, for example, pyogenic bacteria, viral infections, fungi, sarcoidosis, and autoimmune responses [52].

ME/CFS, however, is a chronic disease, which is accompanied by an inability to generate energy on demand [12]. Mitochondrial dysfunctions and abnormally low ATP and high lactate levels play a role in ME/CFS [12,53-61]. ME/CFS patients display exercise-induced exhaustion much earlier than healthy controls. When reaching exhaustion, ME/CFS patients display diminished intracellular ATP and dysregulated oxidative metabolism, coupled to increased glycolysis in the exercising striated muscles [12,58]. ME/CFS patients display reduced rates of ATP resynthesis in the aftermath of exercise versus controls resulting from impaired oxidative phosphorylation [56]. Patients with ME/CFS show prolonged elevations of lactate, returning extremely slowly to normal levels [53,59]. Behan *et al*. [55] found histopathological abnormalities in the mitochondria of skeletal muscles in ME/CFS. During exercise, the latter display decreased voluntary muscle contractions, which worsen subsequently [59]. Thus, in ME/CFS patients reduced intracellular ATP, oxidative metabolism and accelerated glycolysis in skeletal muscles determine early exhaustion [12,58,61]. Several studies report significantly increased levels of ventricular lactate in ME/CFS patients, suggesting central energy dysregulation [62,63].

We previously proposed that the defects in energy production and mitochondrial functions in ME/CFS are probably caused by chronic inflammatory and oxidative and nitrosative stress (O&NS) processes [12,58]. Thus, raised levels of proinflammatory cytokines resulting from systemic inflammation disable oxidative phosphorylation within mitochondria. This is reflected in the increased lactate and mitochondrial dysfunction commonly seen in chronic inflammatory states [64]. For example, TNFα causes a marked decrease in mitochondrial membrane potential, which may increase reactive oxygen species (ROS) [65] and increases mitochondrial membrane permeability leading to membrane depolarization [66]. Increased ROS damages the electron transport chain leading to depleted ATP production, which in turn causes a deficiency in oxidative phosphorylation leading to overt mitochondrial disease [67]. Defects in oxidative phosphorylation leads to increased ROS production, which acts to create self-propagating mitochondrial dysfunction [68] contributing to increased IL-1β and IL-18 via inflammasome induction [69]. Mitochondrial shutdown induced by proinflammatory cytokines is ultimately engineered by nitric oxide (NO) and is reversible [70]. NO signaling regulates mitochondrial number and function [71] and inhibits mitochondrial respiration [72] by competing with oxygen at complex 1 and cytochrome oxidase [73] resulting in diminished ATP production. Peroxynitrite inhibits mitochondrial respiration by modulating electron transport complexes I and III [74]. Attenuation of mitochondrial respiration by for example NO or its derivatives activates ROS and reactive nitrogen species (RNS) produced by mitochondria [75]. In addition, the increased levels of nuclear factor (NF)κB observed in ME/CFS [76] contribute to a shift towards aerobic glycolysis (the Warburg effect) as observed in cancer cells [58].

Kennedy *et al*. [54] reported elevated surrogate biomarkers of O&NS in ME/CFS, which positively associated with symptom exacerbation following energy expenditure. Fatigue results from ROS accumulation and diminished availability of ATP in muscle cells [77]. As to how this relates to the putative subtypes of Booth *et al*. [78], where ME/CFS patients are subdivided on the basis of decreased ADP uptake by mitochondria versus decreased ATP output requires further investigation. These two subgroups are predicted to differentially show increased lactate production and altered ability for repeat exercise. It is not unlikely that changes in oxidative status, lipid peroxidation and mitochondrial membrane rigidity are impacting on the activity of the ADP-ATP translocator (TL), perhaps having differential effects on TL uptake or output functions. Around 20% of mitochondria in cells at any given point in time are in the process of transport, either exhausted and being removed from sites of high-energy need or fresh mitochondria are being imported into such sites. Further investigation is required as to whether the energetic changes in mitochondria in ME/CFS contribute to dysregulated signaling for transport. This may overlap with the genetic susceptibility to ME/CFS mediated by changes in the disrupted-in-schizophrenia 1 (DISC1) gene, which is associated with mitochondrial transport [79]. ATP is also an important neuronal and glia transmitter. As to how altered mitochondrial ATP regulation modulates such wider ATP functions is unknown, but would be expected to contribute to cognitive deficits, especially in high energy demand activities of the ventral lateral prefrontal cortex that are required to unhook specific memory exemplars.

**Figure 1:**
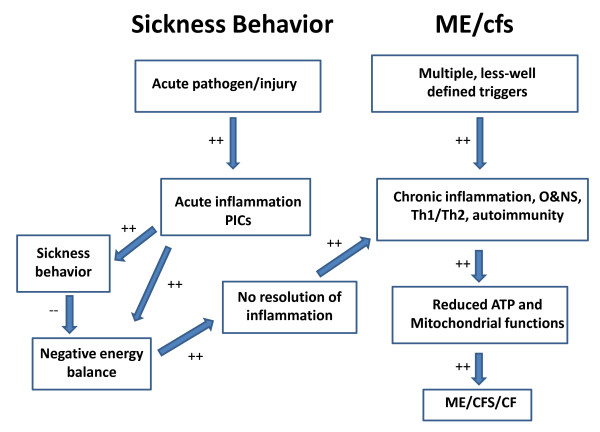
**Summary of differences between sickness behavior and myalgic encephalomyelitis/chronic fatigue syndrome (ME/CFS)**. Sickness behavior is a short-lasting, adaptive and acute inflammatory state induced by acute infection/trauma, while ME/CFS is a disabling chronic disorder associated with multiple, less well defined triggers. While sickness behavior is an acute inflammatory response to proinflammatory cytokines (PICs), ME/CFS is accompanied by a chronic low-grade inflammatory state and a mixed T helper (Th)1 and Th2 response, immune dysfunctions, autoimmune responses and activation of oxidative and nitrosative stress (O&NS) pathways. PIC-induced sickness behavior aims to conserve energy, redirect energy to fuel immunocytes and prevent transition from acute to chronic inflammation. ME/CFS, however, is accompanied by chronic mitochondrial dysfunctions in energy metabolism, for example, reduced ATP production, which are driven, at least in part, by chronic inflammatory and O&NS processes. When there is no resolution of acute inflammation, a chronic inflammatory and thus pathological state may ensue that may lead to the development of ME/CFS some months later.

In summary, energy metabolism plays a key role in sickness behavior and ME/CFS and also differentiates sickness from ME/CFS. Table 1 and Figure 1 show the differences in energy metabolism between both conditions. Thus, sickness behaviors aim to conserve and redirect energy to immune cells, preventing transition from acute to chronic inflammation. ME/CFS, however, shows chronic dysfunctions in ATP and lactate production, caused by peripheral and central mitochondrial dysfunctions, driven, in part, by chronic inflammation and O&NS.

### Immunoinflammatory pathways in sickness versus ME/CFS

Elevated levels of proinflammatory cytokines drive most if not all aspects of the sickness response either directly or indirectly [80]. Proinflammatory cytokines, including IL-1, IL-6, and TNFα, activate the production and/or release of secondary inflammatory mediators, such as prostaglandins (PGs) and NO [81]. Proinflammatory cytokines directly stimulate numerous neurohormonal systems. A variety of mechanisms allow proinflammatory cytokine signals to circumnavigate of the blood brain barrier [82]. Blood borne proinflammatory cytokines can diffuse through relatively permeable areas of the blood brain barrier via the circumventricular organs [6], allowing proinflammatory cytokines to directly interact with microglia and astrocytes in the glia limitans. Another crucial pathway involves a complex series of interactions between proinflammatory cytokines and brain endothelial cells [3]. The third pathway involves the activation of neurons by proinflammatory cytokines via the vagus nerve and catecholaminergic circuits of the sympathetic nervous system (SNS) [12]. (For a more detailed examination of immune to brain communication, see [83].) Interestingly, the production of TNFα persists in the brain much longer than in the periphery [84-86], while TNFα additionally passes both ways across the blood-brain barrier [87]. This is concordant with chronic neuroinflammation and low-grade peripheral inflammation being interconnected phenomena. Within the brain, proinflammatory cytokines, prostaglandins and NO invoke brain responses to infection in the periphery [88]. Once microglia are activated, astrocytes are recruited leading to further activation of neuroinflammatory signals [89]. Thus, both cell types collaborate to propagate neuroinflammation and the physiological changes directed at diminishing the replication of pathogens increasing the metabolism of carbohydrate fat and protein [90] and generating energy-conserving behaviors [3].

The central nervous system (CNS) modulates the immune response via two pathways: (a) the hypothalamic-pituitary-adrenal (HPA) axis and (b) the release of catecholamines and acetylcholine [91]. Communication of proinflammatory cytokine signaling to the brain via the vagus provokes a rapid anti-inflammatory response through increased HPA axis and cholinergic nerve activity [92]. This forms the basis of the CIRS response including the HPA axis, cholinergic and anti-inflammatory responses [3]. This CIRS response in the CNS and autonomous nervous system generates its own characteristic symptom pattern (for example, autonomic symptoms) and is thus part of the sickness response. The SNS modulates the immune response at systemic, local and regional levels [93]. Catecholamines inhibit production of proinflammatory and T helper (Th)1 cytokines, while enhancing the synthesis of Th2 cytokines together with IL-10 and transforming growth factor (TGF) 1 [94]. Acetylcholine release through activation of the parasympathetic nervous system attenuates the production of IL-1, TNFα and IL-6 [91].

Such mechanisms underpinning the sickness response reveals it as an automated irresistibly integrated response conserved by evolution and which is induced by proinflammatory cytokines, including IL-1 and TNFα. The relation between elevated proinflammatory cytokines and fatigue and fatiguability is well documented [95]. Elevated proinflammatory cytokines are largely responsible for the severe fatigue seen in multiple sclerosis [96], cancer-related fatigue and cognitive symptoms [97], fatigued breast cancer survivors [98], and the debilitating fatigue reported by underperforming athletes [99]. Inhibition of IL-1β is associated with a 50% reduction in fatigue in patients with Sjögren's syndrome [100]. Proinflammatory cytokines and prostaglandin E2 (PGE2) mediate inflammatory hyperalgesia [95] as well as neurocognitive abnormalities leading to problems with concentration and memory [95,101]. Many of these adverse effects especially in the CNS are mediated by IL-1β [102]. The hippocampus is the hub of processes involving learning and memory and is also the region of the brain with the highest levels of IL-1 receptors and is especially vulnerable to the effects of neuroinflammation [103]. While physiological levels of IL-1 are essential in the development of memory and learning, elevated endogenous levels results in neurocognitive dysfunctions and abnormal behaviors [104,105]. IL-1β also mediates the suppression of feeding behavior and appears to regulate lipid metabolism by antagonizing the performance of lipoprotein lipase, which is the principal regulator of lipid store mobilization in the body [106]. IL-1β and NFκB are essential mediators of the anhedonic effects of sickness behaviors [107]. Moreover, IL-1β in concert with TNFα inhibits sexual behavior [108]. TNFα and IL-1β suppress the clock genes that regulate circadian rhythm and hence could influence a veritable myriad of biological functions. The net effect of the action of these cytokines on clock genes is to reduce the metabolic rate and the demand for energy in the form of ATP [15].

In patients with ME/CFS, proinflammatory cytokines, including IL-1β and TNFα, are elevated and are significantly associated with the severity of fatigue, a flu-like malaise, sadness and autonomic symptoms [95]. This together with the findings that these proinflammatory cytokines may cause similar sickness behaviors suggest that increased levels of proinflammatory cytokines may contribute to selected ME/CFS symptoms [95]. However, in ME/CFS activation of immunoinflammatory pathways and intracellular signaling networks, including NFκB, inducible nitric oxide synthase (iNOS) and cyclooxygenase 2 (COX-2) and CMI pathways contribute to fatigue, malaise, hyperalgesia, and autonomic and neurocognitive symptoms [12,13,95]. Moreover, there are many more immunoinflammatory related differences between sickness behavior and ME/CFS, including the following.

(1) Elevated levels of proinflammatory cytokines may be associated with a number of other biological disorders that are observed in ME/CFS. For example, as explained in the previous section, TNFα affects energy metabolisms and leads to defects in mitochondria. TNFα also reduces the number of mitochondrial cristae and inhibits mitochondrial respiration. TNFα causes opening of mitochondrial permeability transition pores (PTP) leading to a disappearance of the inner membrane potential and the uncoupling of oxidative phosphorylation [12]. Proinflammatory cytokines also induce O&NS pathways and by generating an increase in ROS in the electron transport chain deplete glutathione [109]. Proinflammatory cytokine elevations in general lead to the upregulation of NADPH oxidase [110], generating superoxide anions and reactive oxygen dependent damage, ultimately leading to the formation of peroxinitrite and membrane damage by lipid peroxidation [111,112]. IL-6 also dramatically increases the rate of glucose usage in striated muscle [113].

(2) Numerous immunoinflammatory abnormalities are also seen in people with ME/CFS indicating an activated but dysregulated immune system, including high levels of serum neopterin [13,95], which is a surrogate marker for increased interferon (IFN)γ levels [114]. High neopterin concentrations occur in diseases with activated Th1 type immune responses, including viral infections, a number of autoimmune diseases and several neurodegenerative diseases [115]. Elevated IFNγ levels deplete tryptophan in the plasma and the brain via activation of indoleamine-2,3-dioxygenase as can be observed in chronic active viral infection [116].

(3) It is worth emphasizing at this point that much of the symptoms and pathology driven by proinflammatory cytokines occurs transiently in sickness behavior, but occurs chronically in ME/CFS. Far from being a Th2 dominated illness it is now clear that Th1 and Th2 cytokines coexist in ME/CFS [12,58]. Moreover, the cytokine profile in ME/CFS patients changes markedly over time [117]. Chronic elevation of Th2 and Th1 cytokines may conspire to create additional and more complex pathologies, for example, accelerating the rate of glucose homeostasis in the brain [118]. Other well documented immune abnormalities in ME/CFS, which are not germane to sickness behavior, include dysregulated forkhead box P3 (FoxP3) expression [119], disrupted T cell homeostasis as indicated by reports of increased CD26 expression [120], decreased expression of CD69 [121] and elevated B cell numbers [122].

(4) A plethora of other immune abnormalities have been detected in patients with ME/CFS, which when taken together demonstrate the existence of a dysregulated immune system. These findings include reduced natural killer cell function and T cell exhaustion [12,58,119-122]. T cell exhaustion and the Th2 response may suggest that ME/CFS is accompanied by activation of the CIRS, as can be observed in many other inflammatory disorders [3,12].

In summary, there is some evidence that shared inflammatory pathways (that is, increased proinflammatory cytokine levels) underpin sickness behavior and determine part of the symptoms in ME/CFS (see also Table 1). However, while sickness behavior is a beneficial response induced by proinflammatory cytokines to conserve energy and combat pathogens thus preventing transition to chronic inflammation, ME/CFS is a chronic inflammatory disorder accompanied by a combined Th1 and Th2 response and multiple signs of a seriously dysregulated immune system.

### O&NS pathways in sickness versus ME/CFS

Other pathways that discriminate sickness behavior from chronic inflammatory disorders are reduced antioxidant levels and increased O&NS, driving O&NS damage to lipids, DNA, proteins and mitochondria (see above). Inflammatory processes, CMI activation and O&NS processes are inseparably associated. For example, phagocytes and activated M1 macrophages produce large amounts of ROS and RNS [123]. CMI activation produces neopterin, increasing ROS and RNS [124]. O&NS processes also activate the production of proinflammatory cytokines and T cells [125]. Therefore sickness behavior may be accompanied by elevated levels of ROS/RNS, which help to eradicate invading pathogens, that then normalize with the resolution of inflammation.

ME/CFS, however, shows chronically increased ROS/RNS, O&NS processes and O&NS damage, including increased malondialdehyde (MDA), isoprostane, 2,3 diphosphoglyceric acid, 8-OH-deoxyguanosine, protein carbonyls and thiobutyric acid [126-130]. iNOS production is significantly higher in ME/CFS patients versus controls [131]. As discussed in the next section, ME/CFS is accompanied by a chronic hyperproduction of NO [95,126]. Raised oxidative stress levels also occur in response to exercise in ME/CFS [129] potentially explaining one of the mechanisms underlying post-exertion malaise [12]. Skeletal muscle oxidative imbalance contributes to increased muscle fatiguability [130].

Lowered levels of coenzyme Q10, zinc and glutathione have been reported in ME/CFS [126,127,131] with amelioration of oxidative stress occurring during remission [128]. As well as decreased coenzyme Q10 [127], decreased vitamin C and E will impact mitochondrial function, increasing lipid peroxidation. Such effects are potentially prevented by melatonin [132], as shown after strenuous exercise in humans [133]. Maes *et al*. [134] reported significantly increased ω6 lineolic and arachidonic acids in ME/CFS, driving a reduced ω3 to ω6 polyunsaturated fatty acids (PUFAs) ratio versus controls, contributing to inflammation in ME/CFS [135].

Such alterations can generate pathology via a number of mechanisms. Isoprostane, a prostaglandin (PG)F2-like compound, correlates positively and significantly with general ME/CFS disease severity as well as the magnitude of exercise induced disease exacerbation [54]. Elevated MDA promotes phospholipids rigidity, altering membrane fluidity, permeability and transport mechanisms [136]. Importantly, elevated MDA decreases mitochondrial membrane fluidity [137]. A decrease in melatonin, found in some ME/CFS studies, would contribute to increased mitochondrial membrane rigidity [138], as well as reduced natural killer cell activity in ME/CFS [119]. Melatonin also modulates aspects of sickness behaviors [139], inhibiting NFκB induction and microglia activation [140], suggesting that variations in melatonin may confound the comparison of processes in ME/CFS and sickness behavior.

In summary, while sickness behavior is probably accompanied by increased ROS/RNS, which should normalize upon the resolution of inflammation, ME/CFS is accompanied by chronically activated O&NS processes causing chronic damage to lipids, DNA, proteins and mitochondria. While in sickness behavior the putative increase in ROS/RNS would be an adaptive response, increased O&NS largely underpins the pathophysiology of ME/CFS.

### Autoimmune responses in ME/CFS

Autoimmune processes also frequently occur in ME/CFS and are not germane to a beneficial short-term response as sickness behavior. IgM autoimmune responses directed against oxidatively damaged lipid membrane components (for example, oleic acid), anchorage molecules (for example, palmitic and myristic acid and *S*-farnesyl-L-cysteine), residue molecules of lipid peroxidation (for example, azelaic acid and MDA), and amino acids or proteins modified by nitrosylating species (for example, NO-tyrosine, NO-phenylalanine, NO-tryptophan, NO-arginine, and NO-cysteine) have all been reported [12,126]. These reactions are directed against neoantigenic determinants (neoepitopes), which are created as a result of damage to lipids and proteins by O&NS [12,126,141]. These antigenic structures are normally invisible to the immune system but become targeted because structural modifications induced by elevated O&NS have rendered them immunogenic. The levels of these corrupted entities correlate positively and significantly with the severity of selected ME/CFS symptoms, such as fatigue, muscle pain, a flu-like malaise, and so on [12,126].

Reports of frank autoimmune reactions are commonplace in people with ME/CFS. Antibodies to cardiolipin, nuclear envelope antigens and neuronal antigens have all been reported [12,142-145]. Several teams have reported elevated titers of autoantibodies directed towards gangliosides, serotonin, phospholipids, anti-68/48K and microtubule associated proteome as well as anti-lamin single stranded DNA [145-147]. Autoantibodies against muscarinic cholinergic receptors, mu-opioid receptors and dopamine receptors have been detected [148]. Autoantibodies directed against ganglioside M1, for example, play a role in neuroimmune disorders, correlating with neurocognitive dysfunctions as observed in neuropsychiatric systemic lupus erythematosus [149,150].

### HPA axis function in sickness and ME/CFS

Sickness behaviors induced by different challenges and in different species are accompanied by marginally increased to significantly increased plasma corticosterone levels [151]. Moreover, corticosterone causes sensitization of LPS-induced pyrexia and pain, but not lethargy, and leads to enhanced sickness-induced neuroinflammation [152]. IL-1, via upregulation of prostaglandin and COX enzymes, enhances HPA axis activation [153] and hypothalamic norepinephrine synthesis [154]. IL-1β also increases brain tryptophan concentrations and the rate of 5- hydroxytryptamine (5-HT) metabolism, which may further activate the HPA axis [154]. Given glucocorticoid anti-inflammatory effects, HPA axis activation is part of the CIRS response [3]. Glucocorticoids also significantly regulate immune genes and cell functions, for example, decreasing the Th1/Th2 ratio [155]. Moreover, glucocorticoids participate in programmed cell death and energy-related processes, for example, glucose, lipid, protein and carbohydrate homeostasis [155,156].

The activation of the HPA axis in sickness behavior contrasts the findings in ME/CFS. HPA axis hypoactivity is a characteristic feature in some people with ME/CFS [157,158], for example, low baseline levels of HPA axis hormones, aberrant diurnal hormone levels, reduced HPA axis responses to provocation by corticotropin-releasing hormone (CRH) or adrenocorticotropic hormone (ACTH), blunted HPA axis responses to physical and psychological stress, and enhanced sensitivity to glucocorticoids [159-165]. This lowered HPA activity may be explained by prolonged stimulation of O&NS and immunoinflammatory pathways in ME/CFS. For example, chronic elevations of IL-6 may blunt the release of ACTH [166]. TNFα may inhibit the stimulation of CRH, ACTH-induced cortisol release and adrenal gland function [167,168].

In summary, while sickness behavior is accompanied by HPA axis activation as part of a CIRS, ME/CFS is accompanied by lowered HPA axis activity, which may be secondary to activation of immunoinflammatory and O&NS pathways (see Table 1).

### Leptin: sickness versus ME/CFS

Leptin is an important mediator of infection-induced inflammation and sickness behaviors [169,170]. However, leptin also accelerates the recovery from hypoxia-induced sickness behavior via an increase in IL-1 receptor antagonist (IL-1RA) [171]. Obese rodents show an altered inflammatory and behavioral response to infection [172].

Treatment of ME/CFS with low dose hydrocortisone increases leptin, leading to a favorable treatment response [173]. Interestingly, recent data shows leptin as a risk factor for, and displaying efficacy in the treatment of depression, as well as Alzheimer's disease [174-176]. Leptin, like melatonin, is a wide immune regulator, increasing natural killer cell activity and Th1 responses, while inhibiting the cAMP induction of tryptophan 2,3-dioxygenase (TDO) in astrocytes, thereby inhibiting cognitive deficits mediated by TDO induction of kynurenic acid. The severity of ME/CFS is associated with increased indicants of metabolic syndrome [177], suggesting that wider metabolic dysregulation associated with obesity and leptin resistance will modulate the course and perhaps etiology of ME/CFS. Thus, leptin may have significant and differential regulatory effects in ME/CFS and sickness behaviors.

### Trigger factors in sickness behavior and ME/CFS

Sickness behavior is an adaptive CIRS response induced by acute injuries or bacterial and viral infections. These phenomenological experiences combined with pyrexia and neuroendocrine changes represent an integrated hierarchal system conserved by evolution to combat infection, conserve energy, play a role in the resolution of inflammation and limit an overzealous inflammatory response [3]. When resolution of inflammation is not induced or when the inflammatory response is overzealous, a chronic inflammatory state may emerge [52,178]. The consequent chronic inflammatory state is then localized where the trigger, either infection or injury, was present leading to persistent infections and medical disorders related to the affected organs or systemic immunoinflammatory and O&NS responses [3,179].

In contrast to the role of acute infection/injury in sickness behavior, different trigger factors such as acute and chronic infections, environmental factors and other medical disorders may play a role in ME/CFS. Depending on the applied case definitions, the presence of medical illnesses may sometimes be regarded as exclusion criteria for a diagnosis of CFS [16]. Thus, Fukuda's criteria consider that the fatigue should not be caused by any conditions that may be identified by specific tests or diagnoses [16]. Other criteria however make the presence of neuroendocrine symptoms and intolerance of mental or physical exercise mandatory [10]. The latter authors propose that a strategy be developed which could detect patients with a neuroimmune condition rather than focus on patient populations that have fatigue whose origin is not revealed by routine medical testing or explained by any psychiatric condition. Moreover, we have argued that ME/CFS may be caused by other inflammatory disorders, including multiple sclerosis, autoimmune disorders, and so on, and that in these conditions also the diagnosis ME/CFS or ME/CFS due to general medical condition should be made [12,17].

Upper respiratory system and flu-like infections often precede the onset of ME/CFS [180]. ME/CFS often occurs in epidemics suggesting that infections may cause ME/CFS [181]. Bacterial and viral infections, including Epstein-Barr virus (EBV), *Coxiella burnetii*, Parvo B19 and *Mycoplasma*, are well known trigger factors associated with the onset of ME/CFS [182]. However, infections not only trigger ME/CFS but may also function as maintaining factors. Thus, many patients with ME/CFS have persistent, recurrent or opportunistic bacterial and viral infections [183,184]. These infections may maintain ME/CFS or cause relapses [185]. Moreover, the number and severity of symptoms, including neurological symptoms, is correlated with the existence of concurrent infections [186,187]. Gene expression data show latent viral or bacterial infections in ME/CFS, for example, Epstein-Barr virus, enteroviruses and *C. burnetii *[188-190]. Other infections that are associated with this disorder are, among others, human herpesvirus (HHV) 6 and 7, cytomegalovirus, enteroviruses, Borna disease virus, *Chlamydia pneumoniae *and *Borrelia burgdorferi *[182].

In summary, sickness behavior and ME/CFS are related to infections. One major difference, however, is that sickness behavior is a short-lasting, adaptive response to acute infection, whereas recurrent or opportunistic infections play a role in the severity and in the relapsing and chronic course of ME/CFS. While sickness behavior is caused by acute infections, the onset of ME/CFS may sometimes be associated with the long-term effects of an acute infection, such as long-standing neuroinflammation, O&NS processes or the onset of autoimmune reactions. Moreover, it is probable that when an initial infection is not cleared and/or there was no resolution of inflammation, a chronic infection with activation of immunoinflammatory and O&NS processes may ensue. Arguably these processes may be associated with the onset of ME/CFS. By inference, in some patients who initially showed sickness behaviors in response to an acute infection, ME/CFS may develop. From a clinical point of view, the transition from acute (sickness behavior) to chronic inflammation occurs between 19 to 43 days. According to the diagnostic criteria of ME and CFS [10,16], the patient cannot be diagnosed as having ME/CFS until 6 months after the onset of the disease. This means that patients, even when experiencing chronic inflammatory processes and from ME/CFS symptoms, cannot be diagnosed as ME/CFS between days 19 to 43 and 6 months. Prolonged or persistent sickness behaviors for this period are not adequate labels because sickness behavior denotes an adaptive and acute inflammatory response, whereas the patients after 19 to 43 days are already in a chronic inflammatory state. Therefore, we propose that those patients should be categorized as having chronic fatigue (CF), a diagnosis which then should be changed into CFS or ME some months later [11].

In contrast to sickness behavior, the initial trigger in ME/CFS is not always well defined, while the chronic stage is associated with positive feedback loops between inflammatory processes and degenerative and autoimmune processes [95,189]. Thus, CFS and ME show a similar pattern to other chronic degenerative disorders in which the initiating trigger is not well defined, for example, clinical depression, schizophrenia, multiple sclerosis, Parkinson's disease, inflammatory bowel disease, cancers, including breast cancer, and autoimmune disorders, lupus erythematosus, Sjögren's syndrome and rheumatoid arthritis, and so on. Moreover, patients with these disorders are primed to develop CF and CFS (and probably ME) through activated immunoinflammatory and O&NS pathways [12,95]. For example, the prevalence of CF/CFS is very high in the above-mentioned autoimmune (41% to 81%) and inflammatory disorders, such as ankylosing spondylitis, biliary cirrhosis, post-poliomyelitis and psoriatic arthritis (48% to 50%) [191]. In post-stroke patients, fatigue is associated with inflammatory biomarkers [192]. Pascoe *et al*. [193] reviewed that post-stroke depression is caused by inflammatory and O&NS processes. By inference, similar processes may explain the onset of post-stroke fatigue. Research also showed a significant association between fatigue and cardiovascular disease (CVD), another inflammatory and O&NS disease [194]. There is also an increased incidence of chronic fatigue in hemodialysis patients ('hemodialysis fatigue') [195]. Since hemodialysis is accompanied by inflammatory and O&NS processes, the latter could also explain the increased incidence of fatigue during hemodialysis. In the postpartum period, another inflammatory state, increased levels of fatigue were found with a significant overlap between postnatal fatigue and depressive symptoms [196]. IFNα-based immunotherapy not only causes clinical depression through induction of the cytokine network, but also by an increased incidence of fatigue and CFS-like symptoms [197,198]. Increased bacterial translocation is also associated with the onset of ME/CFS [199]. Loosening of the gut barrier may allow poorly invasive Gram-negative bacteria to translocate from the gut into the mesenteric lymph nodes and sometimes into the blood stream. Once translocated, the LPS is recognized by the Toll-like receptor 4 (TLR4) complex, which primes immune cells and consequently activates inflammatory and O&NS pathways [12,199].

Psychological stressors are also associated with the onset of ME/CFS [200]. Psychosocial stress also increases the frequency of relapses or a general worsening of symptoms [17]. This may be explained by psychological stress leading to elevated levels of proinflammatory cytokines and O&NS processes that may well create the environment that fosters increased disease activity [201].

In contrast to sickness behavior, which is a CIRS response to infection/injury, a number of pathological predisposing factors may increase the vulnerability to develop ME/CFS. For example, IgG subclass deficiencies, vitamin D deficits, immune gene polymorphisms and a lowered ω3/ω6 PUFA ratio are observed in ME/CFS [12,134]. IgG1 and IgG3 subclass deficiencies increase the risk to infections and autoimmune and inflammatory responses and thus to ME/CFS [12,185]. A decrease in vitamin D is associated with ME/CFS, decreasing not only bone density, but also increasing the susceptibility to, and severity of, infections [202], in part via the regulation of NFκB [203]. Genetic polymorphisms in TNFα, IFNγ, IL-17, IL-10 and HLA genes are associated with the onset of ME/CFS [145]. The DISC1 gene is a susceptibility factor for ME/CFS, as well as for schizophrenia and depression [204]. DISC1 has a significant role in early development, including in the regulation of neuronal migration [205]. Given the relevance of altered mitochondrial function to ME/CFS, it is interesting that DISC1 is strongly associated with mitochondria, significantly regulating mitochondrial function and transport [79]. A possible role of early life immune insult has been proposed for ME/CFS, suggesting that the immune system may be primed by prenatal and postnatal immune regulatory events for an altered response to infection [206]. The lowered ω3/ω6 ratio detected in ME/CFS patients predisposes towards inflammatory and autoimmune responses as ω3 PUFAs are strongly anti-inflammatory and ω6 PUFAs proinflammatory [134].

## Conclusions

**Figure 2:**
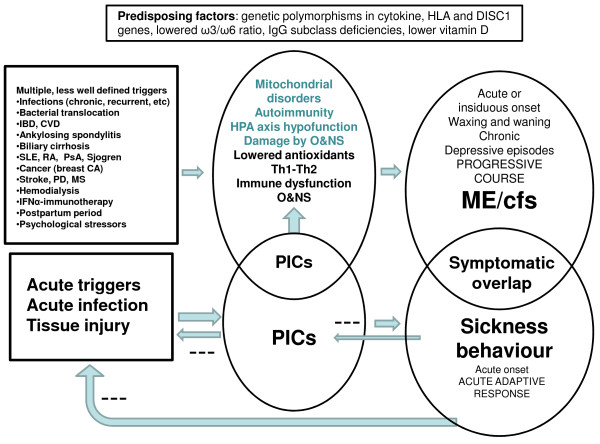
**Comparison of the characteristics of sickness behavior and myalgic encephalomyelitis/chronic fatigue syndrome (ME/CFS)**. While there are significant phenomenological overlaps between sickness behavior and ME/CFS there are major symptomatic differences, such as gastrointestinal symptoms, anorexia, weight loss, psychomotor retardation and pyrexia. Increased levels of proinflammatory cytokines (PICs) may induce the above-mentioned behaviors/symptoms and therefore explain the partial symptomatic overlap between sickness behavior and ME/CFS. While sickness is a short-lasting, beneficial response to acute inflammation, ME/CFS is a disabling disorder with a waxing and waning or progressive pattern and a very low recovery rate. While sickness is a response to acute triggers, the onset of ME/CFS is associated with multiple less well defined trigger factors. While sickness is induced by increased PICs, ME/CFS is accompanied by chronic low-grade inflammation and associated (auto)immune disorders and oxidative and nitrosative stress (O&NS). While sickness is accompanied by activation of the hypothalamic-pituitary-adrenal (HPA) axis, some patients with ME/CFS display HPA axis hypofunction. While acute sickness behaviors aim to conserve energy and prevent transition of acute to chronic inflammation, chronic inflammatory and O&NS pathways conspire to create a state of chronic energy depletion in ME/CFS. In contrast to sickness, predisposing factors increase the vulnerability to develop ME/CFS, including interferon (IFN)γ, interleukin and DISC1 (disrupted in schizophrenia-1) gene polymorphisms. CA = breast carcinoma; CVD = cardiovascular disease; IBD = inflammatory bowel disease; MS = multiple sclerosis; PD = Parkinson's disease; PsA = psoriatic arthritis; RA = rheumatoid arthritis; SLE = systemic lupus erythematosus.

Both sickness behavior and ME/CFS show a phenomenological overlap, both presenting with fatigue, malaise, hyperalgesia, sleepiness, neurocognitive symptoms and mood symptoms. Post-exertional malaise following mental and physical activities, a characteristic symptom of ME, probably also occurs during sickness behavior. While gastrointestinal symptoms are diagnostic criteria for some case definitions of ME/CFS, these symptoms are not typical for sickness behavior. Some typical sickness behavior symptoms, such as psychomotor retardation, anorexia and weight loss do not occur in ME/CFS unless there is comorbid depression. While pyrexia is a hallmark of acute inflammation, mild fever may occur in a few patients with ME/CFS. While sickness behavior is a short-lasting, adaptive and acute inflammatory state, ME/CFS is a chronic disorder characterized by a progressive or relapse-remitting course and with an extremely low recovery rate. While proinflammatory cytokines induce the beneficial sickness behavior response, ME/CFS is accompanied by chronic low-grade inflammation, O&NS, autoimmune responses, and wider immune alterations. Proinflammatory cytokines and Th1 cytokines and other inflammatory mediators together with elevated O&NS can induce a wide range of empirical abnormalities and disabling symptoms. These molecules conspire to create other differences between the acute sickness response, which is a state of proinflammatory cytokine-induced energy conservation, and ME/CFS, which is a chronic disease underpinned by a state of chronic energy depletion [12]. In contrast to sickness behavior, which is a CIRS response to acute infection/injury, the initial trigger in ME/CFS is not always well defined and encompasses acute and chronic infections, multiple autoimmune and (neuro)inflammatory diseases and psychosocial stressors. In contrast to sickness behavior, some specific risk factors predispose to develop ME/CFS, for example, IgG deficiencies, immune gene polymorphisms and reduced ω3/ω6 PUFA ratio. While sickness behaviors are halted with the resolution of inflammation, ME/CFS is a chronic disorder associated with positive feedback loops between inflammatory and O&NS processes as well as degenerative and autoimmune processes. Table 1 and Figure 2 shows that sickness behavior and ME/CFS are two different conditions; that is, an adaptive beneficial response to proinflammatory cytokines on the one hand, and a chronic inflammation/O&NS-related disabling disorder on the other.

## Abbreviations

ACTH: adrenocorticotropic hormone; CF: chronic fatigue; CFS: chronic fatigue syndrome; CMI: cell-mediated immune; CNS: central nervous system; CRH: corticotropin-releasing hormone; CVD: cardiovascular disease; DISC1: disrupted-in-schizophrenia 1; EBV: Epstein-Barr virus; HHV: human herpesvirus; HPA: hypothalamic-pituitary-adrenal; IBS: irritable bowel syndrome; IL: interleukin; IL-1RA: IL-1 receptor antagonist; LPS: lipopolysaccharide; MDA: malondialdehyde; ME: myalgic encephalomyelitis; NF: nuclear factor; O&NS: oxidative and nitrosative stress; PEM: post-exertion malaise lasting more than 24 h; PGs: prostaglandins; PUFAs: polyunsaturated fatty acids; RNS: reactive nitrogen species; ROS: reactive oxygen species; SF-36: 36-item short-form health survey; SNS: sympathetic nervous system; TDO: tryptophan 2,3-dioxygenase; TGF: transforming growth factor; Th: T helper; TNF: tumor necrosis factor.

## Competing interests

No specific funding was obtained for this specific review. MB has received grant/research support from the NIH, Cooperative Research Centre, Simons Autism Foundation, Cancer Council of Victoria, Stanley Medical Research Foundation, MBF, NHMRC, Beyond Blue, Geelong Medical Research Foundation, Bristol Myers Squibb, Eli Lilly, Glaxo SmithKline, Organon, Novartis, Mayne Pharma and Servier, has been a speaker for Astra Zeneca, Bristol Myers Squibb, Eli Lilly, Glaxo SmithKline, Janssen Cilag, Lundbeck, Merck, Pfizer, Sanofi Synthelabo, Servier, Solvayand Wyeth, and served as a consultant to Astra Zeneca, Bristol Myers Squibb, Eli Lilly, Glaxo SmithKline, Janssen Cilag, Lundbeck and Servier. The other authors declare that they have no competing interests.

## Authors' contributions

GM and MM participated in the design of this review, while all authors helped to draft the paper. All authors contributed equally to this paper. All authors read and approved the final version.

## Pre-publication history

The pre-publication history for this paper can be accessed here:

http://www.biomedcentral.com/1741-7015/11/64/prepub
